# Molecular phylogeny of diplomonads and enteromonads based on SSU rRNA, alpha-tubulin and HSP90 genes: Implications for the evolutionary history of the double karyomastigont of diplomonads

**DOI:** 10.1186/1471-2148-8-205

**Published:** 2008-07-15

**Authors:** Martin Kolisko, Ivan Cepicka, Vladimir Hampl, Jessica Leigh, Andrew J Roger, Jaroslav Kulda, Alastair GB Simpson, Jaroslav Flegr

**Affiliations:** 1 Department of Biology, Dalhousie University. Life Sciences Centre, 1355 Oxford Street, Halifax, NS, B3H 4J1, Canada; 2 Department of Biochemistry and Molecular Biology, Dalhousie University. Sir Charles Tupper Medical Building, 5850 College Street, Halifax, NS, B3H 1X5, Canada; 3 Department of Zoology, Faculty of Science, Charles University in Prague. Vinicna 7, Prague, 128 44, Czech Republic; 4 Department of Parasitology, Faculty of Science, Charles University in Prague. Vinicna 7, Prague, 128 44, Czech Republic

## Abstract

**Background:**

Fornicata is a relatively recently established group of protists that includes the diplokaryotic diplomonads (which have two similar nuclei per cell), and the monokaryotic enteromonads, retortamonads and *Carpediemonas*, with the more typical one nucleus per cell. The monophyly of the group was confirmed by molecular phylogenetic studies, but neither the internal phylogeny nor its position on the eukaryotic tree has been clearly resolved.

**Results:**

Here we have introduced data for three genes (SSU rRNA, α-tubulin and HSP90) with a wide taxonomic sampling of Fornicata, including ten isolates of enteromonads, representing the genera *Trimitus *and *Enteromonas*, and a new undescribed enteromonad genus. The diplomonad sequences formed two main clades in individual gene and combined gene analyses, with *Giardia *(and *Octomitus*) on one side of the basal divergence and *Spironucleus*, *Hexamita *and *Trepomonas *on the other. Contrary to earlier evolutionary scenarios, none of the studied enteromonads appeared basal to diplokaryotic diplomonads. Instead, the enteromonad isolates were all robustly situated within the second of the two diplomonad clades. Furthermore, our analyses suggested that enteromonads do not constitute a monophyletic group, and enteromonad monophyly was statistically rejected in 'approximately unbiased' tests of the combined gene data.

**Conclusion:**

We suggest that all higher taxa intended to unite multiple enteromonad genera be abandoned, that *Trimitus *and *Enteromonas *be considered as part of Hexamitinae, and that the term 'enteromonads' be used in a strictly utilitarian sense. Our result suggests either that the diplokaryotic condition characteristic of diplomonads arose several times independently, or that the monokaryotic cell of enteromonads originated several times independently by secondary reduction from the diplokaryotic state. Both scenarios are evolutionarily complex. More comparative data on the similarity of the genomes of the two nuclei of diplomonads will be necessary to resolve which evolutionary scenario is more probable.

## Background

Diplomonads and their close relatives – enteromonads, retortamonads and *Carpediemonas membranifera *– are small flagellates that tend to be found in low-oxygen habitats. Recently they were classified within Fornicata (Metamonada, Excavata) [1]. Very recently an additional member of Fornicata, *Dysnectes brevis*, was described [2]. Most diplomonads, all described enteromonads, and all described retortamonads except one are endobionts or parasites of animals, with several causing serious and highly prevalent diseases in fish, domestic animals and man [3].

Diplomonads and their relatives have been interesting for students of the evolution of the eukaryotic cell for several reasons. Firstly, they lack classical mitochondria. Secondly, diplomonads and their relatives branch at the base of the eukaryotic tree in the majority of phylogenies in which eukaryotes are rooted using prokaryotic outgroups [4-6]. Thirdly, diplomonads, but not their relatives, possess a double karyomastigont, in other words, they have two similar or identical nuclei and two flagellar apparatuses per cell [7,8]. In the late 1980s and early 1990s it was widely supposed that the last common ancestor of most or all living eukaryotes was a 'fornicate-like' amitochondriate organism [4,9], and some models even proposed that almost all living eukaryotes were descended from ancestors with a double karyomastigont [10]. The best-studied diplomonad – *Giardia intestinalis *(= *G. lamblia*) was looked to as a model for understanding early eukaryotic cells.

Recent studies, however, have shown that Fornicata are secondarily amitochondriate [11,12], and that many, perhaps all, retain mitochondrion-related organelles. Small double membrane-bounded organelles called 'mitosomes' were identified in the diplomonad *Giardia intestinalis *using antibodies against IscS and IscU (nucleus encoded proteins targeted to the mitochondrion of eukaryotes) and the function of this organelle in iron-sulfur cluster synthesis was demonstrated *in vitro *[13]. Using electron microscopy, a hydrogenosome-like double membrane-bounded organelle was observed superficially in *Carpediemonas membranifera *[14]. Furthermore, the position of Fornicata at the base of rooted eukaryotic trees is now thought by many to be caused by long-branch attraction [15-17]. Some authors, however, have argued that this basal position might nonetheless still be correct [18].

Diplomonads and enteromonads deserve special attention within Fornicata. These two groups were considered as closely related on the basis of ultrastructural studies [3,7,8] and their close affinity was confirmed recently by molecular phylogenetic methods [19]. The morphology of diplomonads is extremely similar to the morphology of enteromonads – the main character distinguishing these two groups is the doubled karyomastigont of diplomonads [3,7]. In a very simplified way, the cell of diplomonads could be described as two enteromonad cells joined together (and conversely the enteromonad cell could be described as half of a diplomonad cell). The most straightforward scenario explaining the evolution of the doubled karyomastigont is that diplomonads arose from enteromonads in a single evolutionary event. Siddall, Hong and Desser [8] proposed a mechanism of double karyomastigont formation from a single karyomastigont ancestor by secondary karyokinesis (mitosis) and mastigont duplication after delay or arrest of cytokinesis (cell division), resulting in a cell with four karyomastigonts. This cell could then have divided into two cells, each with a doubled karyomastigont. However, some other authors consider as plausible the opposite scenario – secondary simplification from the double karyomastigont morphology of diplomonads to the single karyomastigont morphology of enteromonads [1,20].

Our understanding of the internal phylogeny of Fornicata is based to a large extent on molecular phylogenetic studies, especially with the relatively recent addition of several important taxa to the small subunit ribosomal RNA gene database, namely retortamonads [21], *Carpediemonas membranifera *[22], *Dysnectes brevis *one clade of enteromonads [19], the diplomonad *Octomitus *sp. [23] and several new species from the diplomonad genera *Spironucleus *and *Hexamita *[24]. The monophyly of Fornicata is very strongly supported by molecular phylogenetic studies and there is strong support for a position of *Carpediemonas membranifera *and *Dysnectes brevis *at the base of the Fornicata clade [1,2,22,25]. Meanwhile molecular phylogenies almost invariably divide diplomonads into two major clades, Hexamitinae and Giardiinae, that were already recognized on morphological grounds by Kulda and Nohýnková [3,19,21,24], with the former also identified by the synapomorphy of a non-canonical genetic code [26]. Recently the diplomonad *Octomitus *sp. was confirmed as a sister branch of *Giardia *within Giardiinae [23], and at least one enteromonad group was surprisingly shown to fall within Hexamitinae [19]. Nonetheless, our understanding of the relationships amongst Fornicata is incomplete. For example, different analyses of SSU rRNA gene data place retortamonads either as a sister group of the diplomonad-enteromonad clade [23], as predicted by morphology [1,3], or as a sister branch of the *Giardia-Octomitus *clade, thereby making diplomonads appear paraphyletic [19,21,22]. The main problem in resolving the phylogeny of Fornicata is the limited amount of data, both in terms of taxon sampling and the amount of sequence information per taxon. For example, to date only one enteromonad genus has been studied by molecular means, using only a single gene [19], whereas there are three genera of enteromonads already described, which are quite different in morphology, and enteromonads are generally not recovered as a clade in phylogenetic analyses of morphological data [1,8].

In this study we aim to clarify relationships within fornicates, especially among enteromonads and Hexamitinae diplomonads, and to better understand the evolutionary history of single and double karyomastigonts. We introduce several important taxa into our molecular analyses, including ten new isolates of enteromonads that represent at least three genera. We also introduce two protein-coding genes into the analyses, α-tubulin and HSP90.

## Results

### SSU rDNA phylogeny

Our analyses of SSU rRNA genes include the broadest taxonomic sampling of Fornicates examined so far. In addition to previously available data we have included five new isolates of the enteromonad genus *Trimitus *(the previously published strain KRPO3 was shown to be very closely related to other representatives of the genus *Trimitus*, and therefore we consider KRPO3 as a member of *Trimitus*), three new isolates of the genus *Enteromonas*, two isolates of a new enteromonad genus (isolates PSEUD and PYX, manuscript in preparation), an isolate of *Trepomonas steini *(see Additional files 1 and 2), a new isolate corresponding to the morphospecies *Trepomonas agilis *(PPS-6), a novel *Spironucleus *isolate (GEPA2H) and one uncultured eukaryote (CHESI2). We also extended the previously incomplete SSU rDNA sequence for *Spironucleus muris*. *Trimitus *sp. – IT1 and *Trimitus *sp. – KOMPKOJ represent the first observations and isolations of free-living enteromonads reported so far.

**Figure 1:**
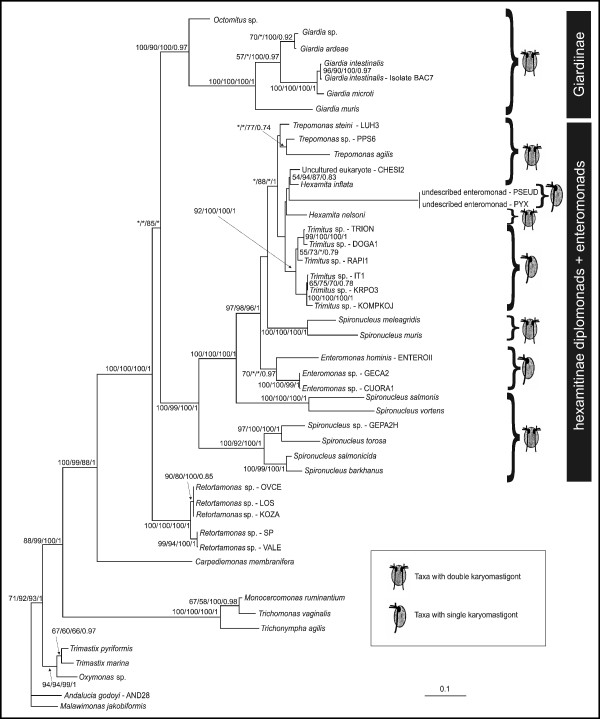
Maximum likelihood tree of Fornicata based on SSU rRNA genes (GTR + Γ + I model). Statistical support – ML bootstraps/RELL bootstraps/ML distance bootstraps/Bayesian posterior probability. Isolate PYX was identical in sequence with isolate PSEUD. Isolate PYX was therefore not included in the analysis but added to the tree by hand. Bootstrap support values <50% and posterior probabilities <0.7 are depicted by asterisks, or not shown.

The SSU rDNA analyses were done using three different alignments, 'large' including a broad eukaryotic outgroup, 'main' including all Fornicata and a restricted outgroup, and 'small' including only Hexamitinae diplomonads and enteromonads. Figure 1 shows the ML tree based on SSU rRNA genes, with a restricted outgroup (i.e. the 'main' data set). The overall topology of the SSU rDNA tree is as follows: Fornicata forms a clade with high statistical support (bootstrap support – BS 100/99/88 and bayesian posterior probability – PP 1), enteromonads branch robustly within the Hexamitinae subtree (BS 100/99/100 and PP 1), and *Giardia *and *Octomitus *form the sister clade of the Hexamitinae-enteromonad subtree, thus rendering diplomonads plus enteromonads monophyletic to the exclusion of retortamonads, but usually with low or no statistical support (BS 43/40/85 and 0.43 PP). Very similar results were obtained when a broader outgroup sampling was employed ('large' dataset; statistical support for Fornicata monophyly: BS 98/97/82 and PP 1; for enteromonad-Hexamitinae monophyly: BS 99/98/99 and PP 1; for diplomonad/enteromonad monophyly: BS */*/64, and not recovered in Bayesian analyses).

**Figure 2:**
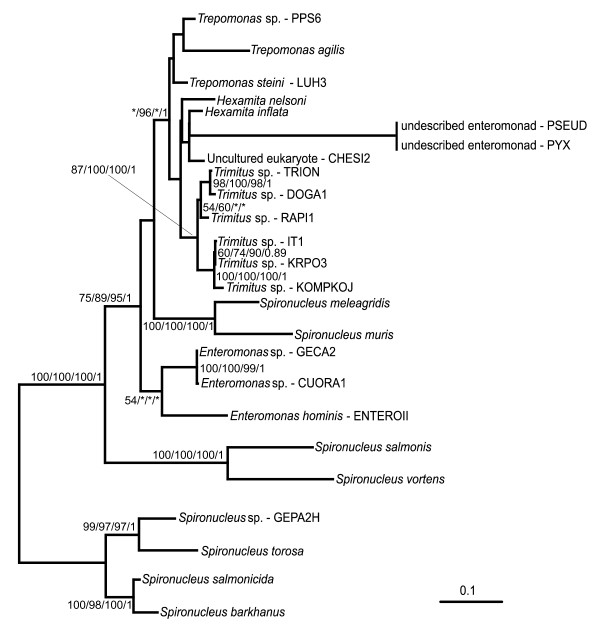
Maximum likelihood tree of the Hexamitinae-enteromonad clade based on SSU rRNA genes (GTR + Γ + I), rooted as per Figure 1. Statistical support – ML bootstraps/RELL bootstraps/ML distance bootstraps/Bayesian posterior probability. Isolate PYX was identical in sequence with isolate PSEUD. Isolate PYX was therefore not included in the analysis but added to the tree by hand. Bootstrap support values <50% and posterior probabilities <0.7 are depicted by asterisks, or not shown.

Given the strong support for a clade consisting of all Hexamitinae and all enteromonads, we performed additional analyses with narrower taxon sampling to examine the internal relationships of this grouping (Figure 2). Within the Hexamitinae-enteromonad clade, members of the genus *Spironucleus *form three separated basal clades in all rooted analyses: i) *Spironucleus vortens *forms a clade with *S. salmonis*; ii) *S. barkhanus *branches with *S. torosa *and *S. salmonicida*; and iii) *S. muris *and *S*. *meleagridis *form a clade (see Figure 1). Our isolate of *Spironucleus*, GEPA2H, branches as a sister of *S. torosa*, suggesting that it could be an isolate of this species, although the genetic distance between GEPA2H and *S. torosa *is greater than that between *S. barkhanus *and *S. salmonicida *(Figure 2). Representatives of the genus *Enteromonas *constitute a weakly supported or unsupported clade in analyses of the main and small datasets, but are polyphyletic in the ML tree estimated for the large dataset, because the isolate *Enteromonas hominis *branches as a sister to *Spironucleus muris *and *S. meleagridis*. The members of the genus *Hexamita *and uncultured eukaryote CHESI2 form a clade with the enteromonads of the genus *Trimitus*, as well as with the new enteromonad genus. All *Trimitus *isolates constitute a highly supported monophyletic group. *Hexamita*, by contrast, does not constitute a monophyletic group within that clade. Genus *Trepomonas *constitutes an unsupported clade (17% ML bootstrap support) in analyses of the main and small datasets and forms a paraphyletic group in analyses of the large data set.

### Concatenation of SSU rDNA, and protein gene data

We have obtained sequences of HSP90 and/or α-tubulin coding genes for all outstanding sampled genera of diplomonads, except *Trepomonas*. For three taxa we obtained only one of the protein coding genes (Table 1). Interestingly, the alternative genetic code usage described earlier in all Hexamitinae (TAA and TAG encode glutamine instead of stop codons; 26), was also identified in the protein coding gene sequences from two out of three enteromonad genera (*Enteromonas *and the undescribed enteromonad genus). Analyses of HSP90 and α-tubulin genes support the monophyly of the genus *Enteromonas*, as *Enteromonas *GECA2 and *Enteromonas hominis *ENTEROII constitute a highly supported clade in both cases (see Additional files 1, 3 and 4).

**Table 1:** Sequences used in our analyses of concatenated genes.

Isolates included in the analyses	Sequenced genes
*Carpediemonas membranifera*	SSU rRNA, HSP90, α-tubulin
*Giardia intestinalis*	SSU rRNA, HSP90, α-tubulin
*Spironucleus vortens*	SSU rRNA, **HSP90**, α-tubulin
*Spironucleus salmonicida*	SSU rRNA, **HSP90**, α-tubulin
*Hexamita inflata*	SSU rRNA, HSP90, α-tubulin
*Spironucleus muris*	SSU rRNA, α-tubulin
*Retortamonas *sp. Vale	SSU rRNA, **HSP90**
*Enteromonas hominis*	**SSU rRNA, HSP90, α-tubulin**
*Enteromonas *sp. – GECA2	**SSU rRNA, HSP90, α-tubulin**
*Trimitus *sp. – TRION	**SSU rRNA, HSP90, α-tubulin**
*undescribed enteromonad*- PSEUD	**SSU rRNA, α-tubulin**

The sequences in bold font were determined during this study.

**Figure 3:**
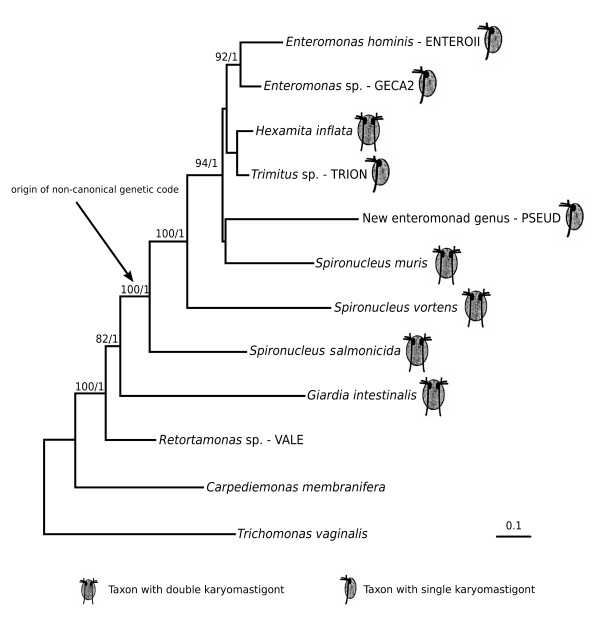
Bayesian tree of concatenated SSU rRNA, α-tubulin and HSP90 genes. Branch lengths shown are those estimated from the HSP90 partition of the concatenated data. Statistical support – 'Bayesian bootstraps'/Bayesian posterior probability.

The phylogenetic tree estimated for the concatenated SSU rDNA, HSP90 and α-tubulin data is shown in Figure 3. As in the SSU rDNA trees, *Spironucleus vortens *and *Spironucleus salmonicida *branch paraphyletically at the base of the Hexamitinae-enteromonad clade with high statistical support. *Spironucleus muris*, the genera *Enteromonas*, *Hexamita *and *Trimitus*, and the new enteromonad genus (represented by isolate PSEUD) constitute a clade with weakly resolved internal relationships. By contrast, *Giardia intestinalis *branches as a sister group of the Hexamitinae-enteromonad clade with high statistical support (BS 82), leaving retortamonads in a sister position to the whole 'diplomonads plus enteromonads' clade.

### AU tests

In order to explore further the evolutionary positions of enteromonads we performed AU tests of alternative topologies. In the case of the SSU rRNA gene data, we compared a set of 1000 reasonable trees, including both the ML tree and the topology of highest likelihood in which enteromonads were monophyletic. For the concatenated dataset, α-tubulin, and HSP90, all reasonable trees were examined (945, 945, and 15 trees respectively, see Methods). The monophyly of enteromonads was not rejected by analyses of any one single gene. In the analysis of the concatenated dataset, however, the monophyly of enteromonads was rejected at the level of 5% (p = 0.048).

## Discussion

### Molecular phylogeny of Fornicata

Our analyses of SSU rRNA genes include the broadest taxonomic sampling of Fornicata published so far. Our results are generally consistent with those of other recent studies [19,21-23]. The genus *Spironucleus *constitutes three separate branches close to the base of the Hexamitinae-enteromonad subtree. This topology is in good agreement with previous studies [21,24,27], however, all three clades of *Spironucleus *constitute long branches and their position at the base of the Hexamitinae-enteromonad subtree could be a long-branch attraction artifact [15]. The presence of *Spironucleus *isolate GEPA2H within the clade of *Spironucleus barkhanus*, *S. torosa*, and *S. salmonicida *is noteworthy, since GEPA2H was isolated from the terrestrial tortoise *Geochelone pardalis*, while the latter three species infect marine teleosts. This argues against Jørgensen and Sterud's [24] proposal that there are more-or-less distinct marine, freshwater, and terrestrial clades within *Spironucleus*. The internal relationships within the remainder of the Hexamitinae-enteromonad subtree were weakly or not supported and vary widely with the method of tree reconstruction and alignment. Further data will be required for a complete picture of the relationships within Hexamitinae.

Analyses of concatenated genes quite strongly support the monophyly of diplomonads plus enteromonads to the exclusion of retortamonads and *Carpediemonas*, albeit within the context of a smaller taxon sampling than is presently available for SSU rDNA alone. This supports previous morphological studies and analyses [1,7,8] but contrasts with some previous studies of SSU rDNA data, in which retortamonads branch weakly within diplomonads, as the sister group to Giardiinae [19,21,22]. The tendency for retortamonads to branch within diplomonads in SSU rDNA analyses is most probably an analysis artifact, but the cause of this artifact is not clear. Our preliminary analyses do not support the notion that either base composition heterogeneity (*Giardia *SSU rDNA is notable for its high GC content, however neither LogDet distance correction nor RY recoding results in a different well resolved topology) or simple long-branch attraction is to blame (data not shown). It is also possible that SSU rDNA simply does not contain enough information to resolve the phylogenetic relationships among the ingroup taxa.

### The positions of enteromonads

Enteromonads and Hexamitinae diplomonads form a monophyletic group to the exclusion of other Fornicata. This is consistent with previous molecular phylogenies that included a much smaller sampling of enteromonads [19] and one morphological study [1]. The clade has very strong statistical support in our analyses, and is further supported by a molecular synapomorphy: the non-canonical genetic code common to all studied Hexamitinae [26] appears also to be present in at least two of the three enteromonads for which we obtained protein-coding gene sequence data.

The most striking result of our study is the non-monophyly of enteromonads. The possibility of enteromonads being polyphyletic was suggested speculatively by Simpson [1] on the basis of morphological data. The internal relationships of the Hexamitinae-enteromonad clade are weakly supported at present, and need further investigation. More extensive taxon sampling for studied protein-coding genes is necessary, as well as more protein-coding genes. Nonetheless, our results support the non-monophyletic status of enteromonads, as the monophyly of enteromonads was rejected by AU test with the concatenated data set. Interestingly, none of the single gene phylogenies themselves rejected enteromonad monophyly. The differing results from analysis of the concatenated dataset and the single-gene datasets could be caused either by an insufficient amount of data in single gene analyses or by conflicting signal between single gene phylogenies. However, none of the single gene ML trees was rejected in AU tests of the concatenated dataset, suggesting that there is no strong conflict between single gene phylogenies. It is reasonable to assume therefore that the rejection of enteromonad monophyly on the basis of concatenated data, but not in to single gene analyses, is due to insufficient signal rather than conflicting single-gene data.

Many authors have treated diplomonads and enteromonads as taxa of equivalent rank [3,7,28], implicitly or explicitly reflecting a widespread assumption that diplomonads and enteromonads are sister clades, or that enteromonads represent a paraphyletic group from which a monophyletic diplomonads group evolved [8,28]. Our analysis demonstrates, however, that many, and probably all enteromonads fall within diplomonads and specifically within Hexamitinae. Moreover, enteromonads represent a non-monophyletic group within Hexamitinae. There is no possible interpretation of our results that would allow recognition of diplomonads and enteromonads as separate taxa, without at least one of them being polyphyletic. Therefore, we suggest that the taxon Diplomonadida and any of its effective synonyms be considered to include enteromonads, and that the taxa Enteromonadida and Enteromonadinae no longer be used. The term 'enteromonads' should be taken to have a purely descriptive, and not taxonomic, meaning.

### Evolution of single and double karyomastigont cell organization

In a previous study [19] it was shown that at least one enteromonad clade branches within diplomonads, and branches with or within the diplomonad subgroup Hexamitinae, suggesting either that enteromonads are secondarily simplified, possibly in one evolutionary event, or that the double karyomastigont morphology characteristic of diplomonads arose more than once during their evolution. Our present study indicates that enteromonads do not constitute a monophyletic group within Hexamitinae, and thus switches between single- and double-karyomastigont morphology must have occurred several times during evolution, irrespective of the direction in which these switches occurred. If we assume only one direction of change within the diplomonad-enteromonad clade and assuming, for argument's sake, that our topologies are correct, there are two basic possibilities: either the double-karyomastigont morphology arose many times in closely related groups (at least seven times according to the SSU rRNA gene tree, or at least five times, according to the multigene analyses) or enteromonads arose by secondary reduction from double-karyomastigont ancestors at least three times independently.

**Figure 4:**
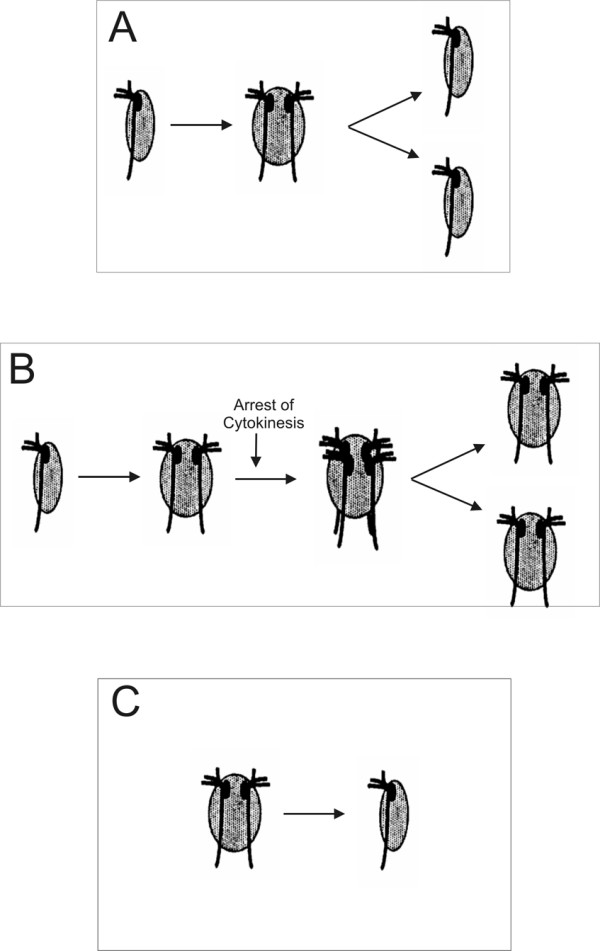
A. "Standard" cell cycle of an enteromonad cell; cell divides after karyokinesis. B. Model of evolutionary change from single karyomastigont morphology to double karyomastigont morphology by arrest of cytokinesis. The cell does not divide after the first karyokinesis and secondary karyokinesis results in a cell with four karyomastigonts. This cell then divides into two cells, each with a double karyomastigont. C. Model of evolutionary change from double karyomastigont morphology to single karyomastigont morphology, either by cytokinesis without karyokinesis, or by fusion of nuclei. (modified from Siddall, Hong and Desser 1992).

There are three potential scenarios describing the switch between single and double karyomastigont morphologies (Fig 4). For comparison, the standard cell cycle of an enteromonad is depicted in Figure 4A.

1. The switch from single karyomastigont morphology to double karyomastigont morphology was envisaged by Siddall, Hong and Desser [8] as a change in the relative timing between karyokinesis and cytokinesis (Figure 4B). Under this model a cell with a single karyomastigont goes through nuclear and mastigont division and is prepared for cell division. Cell division is arrested, however, and the cell goes through another nuclear and mastigont division, resulting in a cell with four karyomastigonts. The cell with four karyomastigonts then divides into two cells, each with a double karyomastigont.

2. The opposite switch (Figure 4C), from a double to single karyomastigont, could also be explained as a change in the timing of karyokinesis and cytokinesis. Cells with a double karyomastigont morphology could just go through cell division without nuclear and mastigont division. Another similar event would involve a cell with four karyomastigonts (i.e. a cell normally with a double karyomastigont prepared for cell division) dividing into four daughter cells instead of two.

3. The last scenario suggests fusion of the nuclei in a cell with the double-karyomastigont morphology. The resulting cell could then lose the second mastigont, resulting in a cell with a single-karyomastigont morphology.

These scenarios have different strengths and weaknesses in terms of plausibility. Scenario one seems implausible from a phylogenetic perspective, as it requires a very large number of parallel evolutions of the distinctive doubled cell morphology within the diplomonad-enteromonad clade. Scenario two invokes many fewer evolutionary events, but requires that the two nuclei of the parental diplomonad cell be nearly identical (or, at a minimum, that at least one of the nuclei retains all essential genes), otherwise the single karyomastigont progeny would not be viable. Scenario three requires the same number of evolutionary transitions as scenario two, and is compatible with a diplomonad parent that had non-identical and essential nuclei, but is otherwise a more complex mechanism. Therefore, the key to understanding the evolution of nucleus number in diplomonads lies in knowing whether the nuclei of diplomonads are identical. Naïvely, it might be expected that the nuclei are non-identical, since one copy of each nucleus is transmitted to each daughter cell during division [29], and, in principle at least, the two nuclei would represent separate lineages once the diplomonad state has been fixed. This would allow essential genes to be lost from one or the other nucleus, such that both nuclei would soon be required for the cell lineage to persist and any uninucleate progeny would be unviable. However, this could be avoided if there were mechanisms that frequently generated cells in which both nuclei were copies of a single parental nucleus (most likely a sexual process).

Empirical data on the nature of diplomonad nuclei are limited and conflicting. The human parasite *Giardia intestinalis *is the only diplomonad whose molecular and cellular biology has been studied in detail. Yu, Birky and Adam [30] used probes against selected genes to indicate that each nucleus of *G. intestinalis *contains a complete set of genetic information. Bernarder, Palm and Svard [31] have also deduced from results of FACS analysis that the two nuclei of *G. intestinalis *are diploid. The genome sequence of *Giardia intestinalis (lamblia) *strain WB clone C6 was published by Morrison *et al*. [32], and minimal heterozygosity was detected. Very recently, a process of physical transfer of DNA between nuclei was reported in *Giardia *cysts [33]. However, Tůmová *et al*. [29] reported that the two nuclei of *G. intestinalis *possess different numbers of chromosomes. The focus on *Giardia *is unfortunate in some respects, as *Giardia *is a highly specialized parasite with an organization of cytoskeletal components (at least) that is substantially different from other diplomonads. It is possible that results obtained for *Giardia *may not be applicable to other diplomonads. *Giardia *also represents the diplomonad group that is most distantly related to the various enteromonad taxa. It would be of great interest to compare the nuclei in one or more Hexamitinae diplomonads (e.g.: *Spironucleus*, *Hexamita *or *Trepomonas*).

There are some observations that would support scenario 1, albeit indirectly. Firstly, the populations of enteromonads often contain some double individuals resembling *Hexamita *in their morphology, with two fully developed karyomastigonts and no apparent signs of cytokinesis [[7,34], M. K. personal observation]. This may suggest that there is some general tendency for delayed or arrested cytokinesis. Secondly, a recent study of the flagellar cycle of *Giardia intestinalis *has shown that the two karyomastigonts are not independent, as basal bodies migrate between the two karyomastigonts [35]. This means that the flagellar maturation cycle would be corrupted in cells that switched back to a single karyomastigont.

Our molecular phylogenies contradict any scenario invoking just one unique evolutionary transition between single and double karyomastigont morphologies within diplomonads. According to the inferred topology, the most parsimonious scenario would have one transition from single to double karyomastigont morphology at the base of diplomonads and several independent reversals to the single karyomastigont morphology. However, taking into account possible inaccuracies in our estimated tree, principally involving *Spironucleus*, and the limited data on diplomonad cell biology, several independent transitions from single to double morphology (and no reversals) cannot be excluded. More data on the molecular and cellular biology of diplomonads in addition to *Giardia *will be necessary for understanding the enigmatic evolution of the double karyomastigont of diplomonads.

## Conclusion

Our analyses of SSU rRNA, HSP90 and α-tubulin genes strongly positioned all enteromonads within Hexamitinae diplomonads and showed that enteromonads do not constitute a monophyletic group. These results suggest that transformations between single- and double-karyomastigont morphologies have occurred several times during the evolution of diplomonads, however, it is not possible to confidently determine the direction of these switches without more information about the cellular and molecular biology of diplomonads and enteromonads. We suggest that the high level taxa Enteromonadida, Enteromonadidae and Enteromonadinae should be abandoned and the genera *Enteromonas *and *Trimitus *should be considered as members of Hexamitinae diplomonads. The term 'enteromonad' should have a purely utilitarian meaning – Diplomonadida with a single karyomastigont.

## Methods

### Cultures

Isolates used in this study are summarized in Table 2. All enteromonad isolates were obtained from animal guts or feces, except isolates KOMPKOJ and IT1, which were free living. *Trepomonas steini *and *Trepomonas *sp.-PPS6 were isolated from anoxic fresh water sediments by the authors. A culture of *Spironucleus vortens *was obtained from the American Type Culture Collection (ATCC #50386). DNA from *Spironucleus muris *was isolated from purified cysts obtained from the intestine of a SCID laboratory mouse. Xenic cultures of enteromonads were grown in Dobell-Leidlaw biphasic medium [36] and in TYSGM medium [37] without tween and mucin at 21°C, 27°C and 37°C. *Spironucleus vortens *was grown axenically in TYI-S33 medium as modified for *Giardia *at 27°C [38]. *Trepomonas *sp.-PPS6 and *Trepomonas steini *were grown in cerophyll medium (ATCC #802) at 21°C (Table 2). DNA from *Spironucleus *sp. GEPA2H and uncultured eukaryote CHESI2 was isolated from crude cultures.

**Table 2:** Diplomonad and enteromonad isolates used in our study for sequencing.

Isolate	Strain	Source	Medium	t (°C)	Other Eukaryotes
*Enteromonas *sp.	GECA2	*Geochelone carbonaria*	Dobell and Leidlaw	27	none
*Enteromonas *sp.	CUORA1	*Cuora amboinensis*	Dobell and Leidlaw	27	none
*Enteromonas hominis*	ENTEROII	*Homo sapiens*	Dobell and Leidlaw	37	none
Enteromonad	PSEUD	*Trachemis scripta elegans*	Dobell and Leidlaw	27	*Retortamonas *sp.
Enteromonad	PYX	*Pyxidea mouhoti*	Dobell and Leidlaw	27	Parabasalids
*Trimitus *sp.	KOMPKOJ	Compost, Kojčice, Czech Republic	TYSGM	21	none
*Trimitus *sp.	IT1	Pond in Italy	TYSGM	21	none
*Trimitus *sp.	DOGA1	*Doagania *sp.	Dobell and Leidlaw	27	none
*Spironucleus vortens*	ATCC#50386	ATCC	TYI	27	none
*Spironucleus *sp.	GEPA2H	*Geochelone pardalis*	Dobell and Leidlaw	n/a	n/a
*Trepomonas steini*	LUH3	Flood, Vltava river, South Bohemia, Czech Republic	Cerophyll	room temp.	*Sawyeria *sp.
*Trepomonas *sp.	PPS6	Point Pleasant Park pond, Halifax, NS, Canada	Cerophyll	21	none
Uncultured eukaryote	CHESI2	*Chelodina *sp.	n/a	n/a	n/a

^1^The SSU rDNA sequence of isolate PYX was identical with isolate PSEUD. Isolate PYX was therefore not included in the phylogenetic analyses.

### Gene amplification and sequencing

Genomic DNA was isolated using a High Pure PCR template kit (Roche Applied Science, UK) or using CTAB and organic extractions [39]. SSU rDNA sequences were amplified by PCR using primers 'EntUnvF' and 'EntUnvR' [19] or universal eukaryotic primers [40]. In the case of the 'new enteromonad genus' isolate 'PSEUD', the culture also contained a retortamonad species. The SSU rDNA segments from both eukaryotes were amplified and partially sequenced. Specific primers for the 'new enteromonad genus', DimA (5'-AGTCAAAGATTAAAACATGCATAT-3') and DimB (5'-TCCTCTAAGCCTTCTAGTTCGTGCAAA-3') were then designed and used for amplification of the SSU rDNA from isolate 'PYX', which is an enteromonad closely related to isolate 'PSEUD'. A specific forward primer (SSUSmur20F 5'-AACTGCGGACGGCTCATT-3') was designed for *S. muris *and used with the universal eukaryotic reverse primer.

Alpha-tubulin genes were amplified using primers AtubA and AtubB [41] and then by nested PCR with primers α-tubF1 and α-tubR1 [42]. HSP90 genes were amplified using primers H90100X [43] and H90910XR [25]. The annealing temperatures used were 45–53°C, 45–50°C and 48–53°C for SSU rDNA, α-tubulin and HSP90, respectively. SSU rDNA amplicons were sequenced directly where possible. Otherwise, major PCR fragments of the expected sizes were subcloned (TOPO TA cloning kit for sequencing, pCR4-TOPO vector, Invitrogen, USA; or pGEM-T Easy vector cloning kit, Promega, USA) and several clones (2–6) were partially sequenced. Obtained sequences were then subjected to BLAST searches [44] to confirm their identity. At least one of the positive clones was fully sequenced bidirectionally by primer walking. All sequences obtained during this study are deposited in GenBank [GenBank: EF551168 – EF551186, EU043230, AY921407 and AY921408].

### Phylogenetic analyses

#### Alignments

All alignments used in this study were constructed using the program ClustalX 1.83 [45] followed by manual editing in the program BioEdit 7.0.5.3 [46] and are available upon request (see Additional files 1 and 5 for details about the sequences used).

#### SSU rRNA genes

Two data sets were constructed including all near-full-length Fornicata sequences, except some redundant close relatives within *Giardia *and retortamonads, plus an outgroup consisting of either i) a broad diversity of eukaryotes (large dataset), or ii) a few supposed close relatives of Fornicata – the excavate groups Parabasalia, *Trimastix*, Oxymonadida, *Malawimonas *and *Andalucia *(main dataset). These datasets included 887 and 1041 well-aligned sites, respectively. An additional dataset was generated that included only Hexamitinae and enteromonads, and also included 1041 sites (small dataset).

Each dataset was analyzed using several likelihood-based phylogenetic methods. The model of sequence evolution was selected by the Akaike information criterion, as implemented in the program Modeltest 3.7 [47]. The general time reversible model of nucleotide substitution was used, with among-site rate variation modeled by a gamma distribution and a proportion of invariable sites (GTR + Γ + I model), with the gamma distribution approximated by 4 equiprobable discrete categories. Maximum likelihood (ML) analyses were performed using the program PAUP*4B10 [48], with 10 random taxon additions followed by tree bisection and reconnection branch rearrangements, while ML bootstrap support (200 replicates) was estimated using PAUP*4B10 (10 random taxa additions followed by TBR; for the large dataset only the ML bootstrap analysis was instead performed using the program IQPNNI 3.0.1. [49]), and LRSH-RELL bootstrapping (1000 replicates) was performed using the program Treefinder (version: February 2007) [50]. The model of sequence evolution used was the same for all ML analyses. Least squares distance trees were estimated from ML distances using PAUP*4B10 and bootstrapped with 1000 replicates (each searched using 10 replicates of random taxon addition with TBR branch swapping). The Bayesian analysis was performed using the program MrBayes 3.1.2 [51], using the GTR + Γ + I model with two runs, each with four independent chains running for 3 × 10^6 ^generations (a burn-in of 5 × 10^5 ^generations was used), with default heating parameter and sampling frequency (which was also used in all subsequent Bayesian analyses).

#### Analyses of protein coding genes

The HSP90 and α-tubulin amino acid datasets included all available Fornicata sequences and an extensive eukaryotic outgroup consisting of representatives from major eukaryotic groups. The trimmed alignments included 370 sites for α-tubulin and 493 sites for HSP90. Both datasets were analyzed using the WAG + Γ + I model [52]. The WAG matrix was selected over other substitution matrices by the Akaike information criterion, as implemented in the program ProtTest 1.4 [53]. For each, the ML tree was estimated and bootstrap support (500 replicates) was estimated using IQPNNI 3.0.1, while LRSH-RELL bootstrap support (1000 replicates) was determined using Treefinder (version: February 2007). In addition, a Bayesian analysis (WAG + Γ + I model) was performed using MrBayes 3.1.2, with four independent chains running for 2 × 10^6 ^generations, and with a conservative burn-in of 5 × 10^5 ^generations.

#### Analyses of concatenated SSU rDNA and protein sequences

The concatenated alignment of SSU rDNA, α-tubulin and HSP90 genes was analyzed using the program MrBayes 3.1.2 (two runs each with four independent chains running for 5 × 10^6 ^generations with a burn-in of 1.5 × 10^6 ^generations), with among-site rate variation for each gene modeled by a discrete approximation of a gamma distribution, proportion of invariable sites and a covarion model. The GTR substitution model was used for the SSU rDNA partition and the WAG substitution matrix [52] was used for the protein coding genes. The branch lengths, α parameter, proportion of invariable sites and parameter for switching rates in the covarion model were estimated separately for each gene (both runs converged to the same level). The branch lengths in the depicted tree are those estimated for the HSP90 partition of the data (The alternative of displaying the average of the estimated branch lengths over all three genes was not followed on the grounds that this average does not reflect any actual parameter examined under the model of evolution we used). In addition to examining posterior probabilities we performed a full bootstrap analysis with 100 replicate samples. Each gene was re-sampled independently (using the program Seqboot from the Phylip package [54]) and then each bootstrap sample was created by concatenating one replicate from each gene. Each bootstrap replicate was analyzed under the same conditions as the starting dataset but using only 2.5 × 10^5 ^generations (a burn-in of 50000 generations was used). The consensus tree was made for each bootstrap replicate in MrBayes 3.1.2. The bootstrap consensus tree was then estimated from the 100 resulting trees using the program Consense from the package Phylip 3.67 [54].

### Testing of topologies

The topologies were compared using 'Approximately Unbiased' (AU) tests implemented in the program Consel 1.19 [55]. We performed separate AU tests on four datasets – 1. SSU rRNA genes, 2. α-tubulin, 3. HSP90, and 4. the concatenated dataset (with the missing genes treated as missing data). For the SSU rRNA test, the small dataset, which includes only Hexamitinae and enteromonads was used. For the α-tubulin, HSP90 and combined datasets, the alignments used for estimating the ML tree were used, except that taxa other than Hexamitinae and enteromonads were excluded. For AU tests using the SSU rRNA gene data we generated a set of ,reasonable trees'. This set was generated by saving the 999 trees with the highest likelihood found during ML analyses in PAUP*4B10 (10 random sequence additions plus TBR). The tree representing monophyletic enteromonads was generated using a constraint search in PAUP*4B10 (10 random addition replicates plus TBR). In the case of α-tubulin, HSP90, and concatenated analyses, we included all possible trees that were consistent with a constraint where nodes corresponding to those that had received 100% bootstrap support in the concatenated genes analysis were fixed. Site likelihoods were calculated using PAUP*4B10 for the SSU rRNA gene data, and using the PAML package [56] for protein data. For the concatenated gene analysis site likelihoods were generated separately for all three genes and then concatenated prior to analysis in Consel 1.19.

## Authors' contributions

MK contributed by gene amplification and sequencing (SSU and HSP90), data analyses and by drafting the manuscript. IC isolated most of the new strains examined in this study, and obtained α-tubulin sequences. VH contributed to data interpretation and by critical reading of the manuscript. JL contributed to data analyses and by critical reading. AJR contributed by critical reading and by supervision of JL and MK. JK contributed by critical reading and isolation of isolate ENTEROII. AGBS contributed to data interpretation, writing the manuscript and by supervising MK. JF contributed to data interpretation, writing the manuscript and by supervising MK at Charles University, Prague.

## Supplementary Material

Additional file 1 Supplementary materials. Includes additional table and figures description. Click here for file

Additional file 2 Supplementary materials – Figure 1. Includes additional figure. Click here for file

Additional file 3 Supplementary materials – Figure 2. Includes additional figure. Click here for file

Additional file 4 Supplementary materials – Figure 3. Includes additional figure. Click here for file

Additional file 5 Supplementary materials – Table 1. Includes additional table. Click here for file
