# Supplementary material for: Molecular phylogeny of diplomonads and enteromonads based on SSU rRNA, alpha-tubulin and HSP90 genes: Implications for the evolutionary history of the double karyomastigont of diplomonads
Source: BMC Evol Biol. 2008 Jul 15;8:205. doi: 10.1186/1471-2148-8-205 (PMC2496913; doi:10.1186/1471-2148-8-205)
Supplement: Additional file 1 — Supplementary materials. Includes additional table and figures description. [file 1471-2148-8-205-S1.pdf]

Additional files

Additional file 2. DIC light microscopy photographs of *Trepomonas steini*. Two quite long flagella are directed laterally while two posterior flagella extend beyond the end of the cell (four shorter flagella do not extend past the posterior end of the cell), which is typical for *T. steini*. As is typical for *T.steini*, the cell moves in one of two alternating modes i) slowly with a regular jerky rotation around the longitudinal axis, ii) a faster smooth movement. Scale bar is 10 µm.

Additional file 3. Maximum likelihood (WAG + Γ + I) tree of Fornicata based on HSP90 protein sequences. Statistical support is as follows IQPNNI 500 bootstraps / 1000 RELL bootstraps / Bayesian posterior probability. * means that branch was not recovered in the majority rule consensus tree of the bootstrap analyses. Only statistical support within Fornicata shown.

Additional file 4. Maximum likelihood (WAG + Γ + I) tree of Fornicata based on tubulin protein sequences. Statistical support is as follows IQPNNI 500 bootstraps / 1000 RELL bootstraps / Bayesian posterior probability. Only statistical support within Fornicata shown.

Additional file 5. Table of all sequences used for analyses.
