# Supplementary figures and images for: Molecular phylogeny of diplomonads and enteromonads based on SSU rRNA, alpha-tubulin and HSP90 genes: Implications for the evolutionary history of the double karyomastigont of diplomonads
Source: BMC Evol Biol. 2008 Jul 15;8:205. doi: 10.1186/1471-2148-8-205 (PMC2496913; doi:10.1186/1471-2148-8-205)

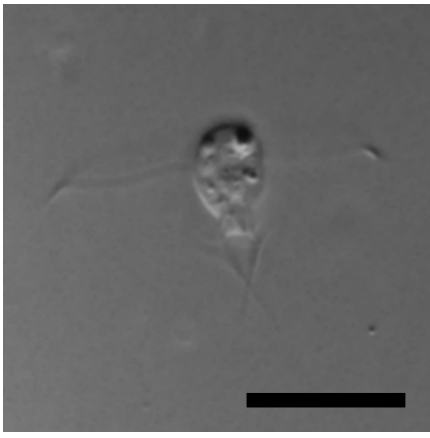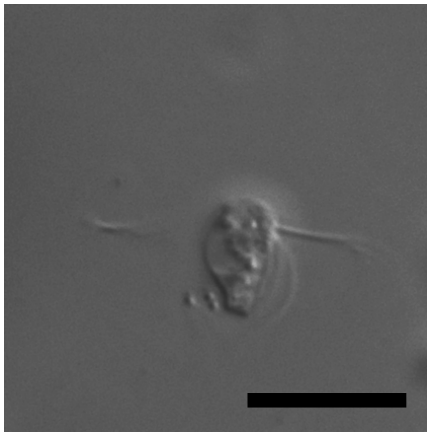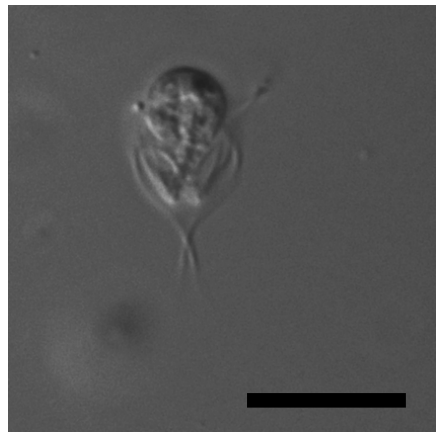

Supplement: Additional file 2 — Supplementary materials – Figure 1. Includes additional figure. [file 1471-2148-8-205-S2.pdf]

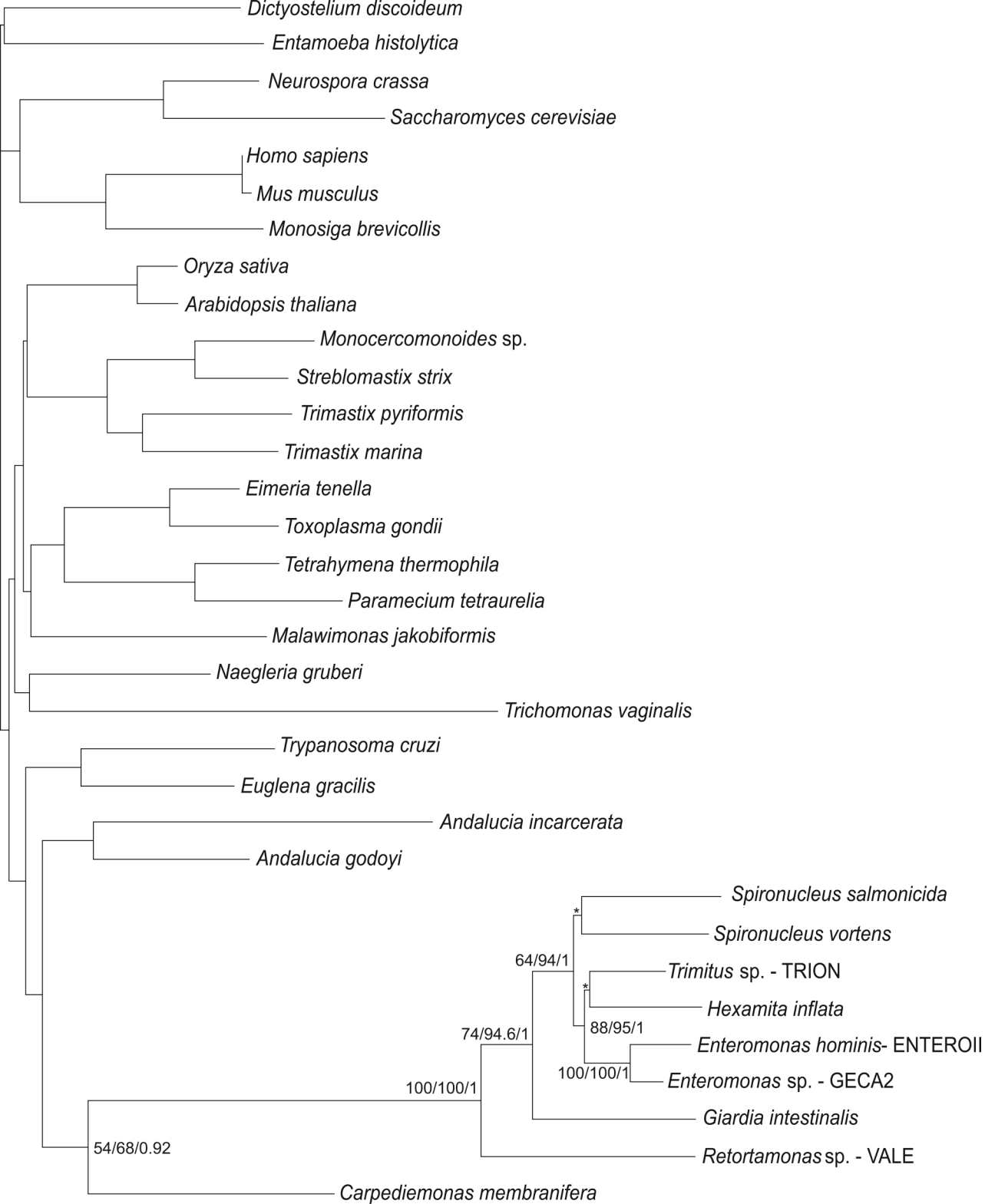

Supplement: Additional file 3 — Supplementary materials – Figure 2. Includes additional figure. [file 1471-2148-8-205-S3.pdf]

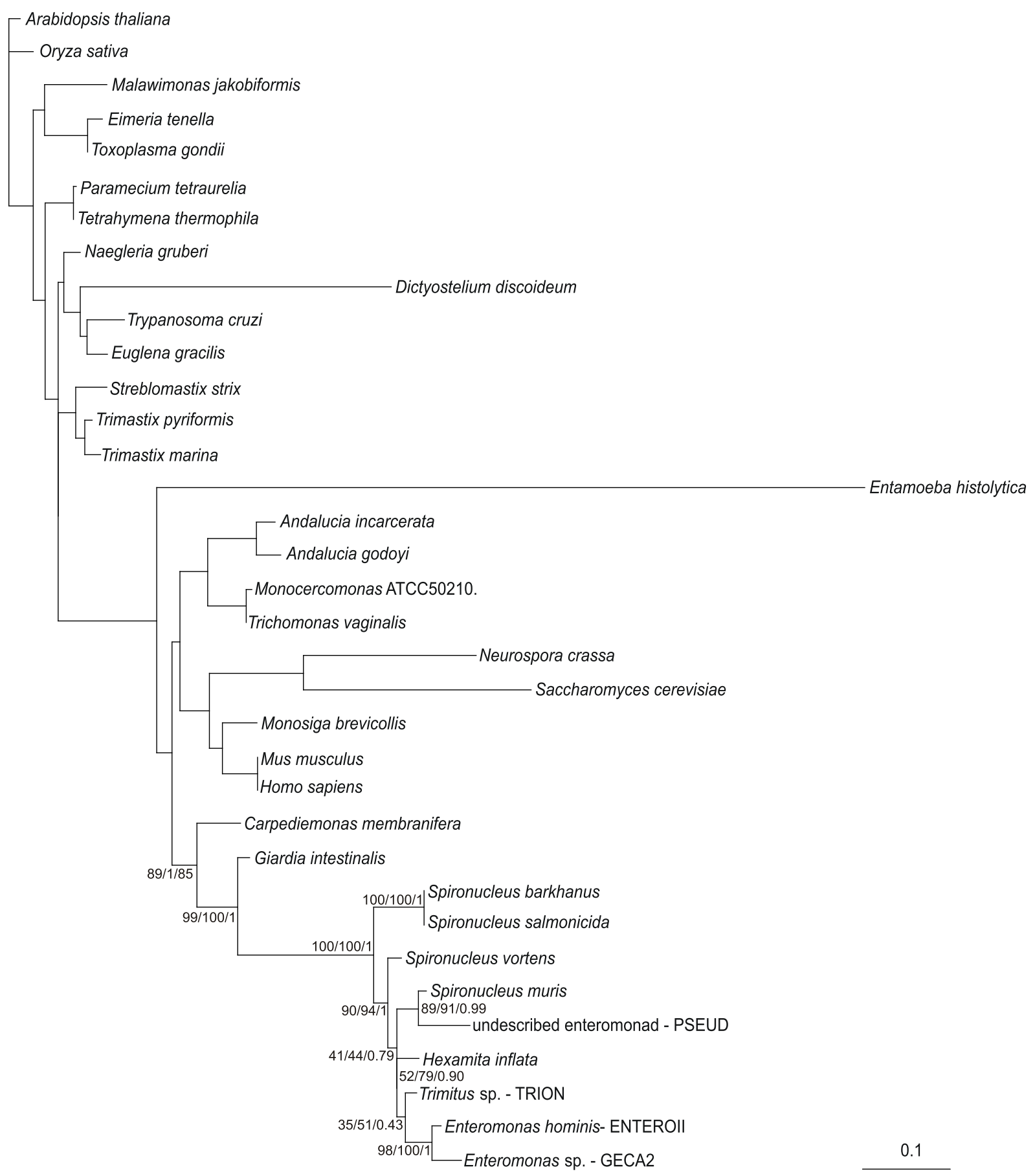

Supplement: Additional file 4 — Supplementary materials – Figure 3. Includes additional figure. [file 1471-2148-8-205-S4.pdf]
