# Supplementary material for: Molecular phylogeny of diplomonads and enteromonads based on SSU rRNA, alpha-tubulin and HSP90 genes: Implications for the evolutionary history of the double karyomastigont of diplomonads
Source: BMC Evol Biol. 2008 Jul 15;8:205. doi: 10.1186/1471-2148-8-205 (PMC2496913; doi:10.1186/1471-2148-8-205)
Supplement: Additional file 5 — Supplementary materials – Table 1. Includes additional table. [file 1471-2148-8-205-S5.pdf]

Taxon	Isolate	SSU rRNA gene	α-tubulin	hsp90
Acanthometra sp.		AF063240.1		
Acrasis rosea		AF011458.1		
Andalucia godoyi AND28		AY965870.1	EU334874	EU334880
Andalucia incarcerata			AAK27844.1	EU334886
Arabidopsis thaliana			NP_193232.1	AAN31859
Carpediemonas membranifera		AY117416.1	AAM77193.1	ABC54644
Cercomonas ATCC50317		U42449.1		
Clathrulina elegans		AY305009.1		
Cryptosporidium parvum		L16996.1		
Cyanophora paradoxa		X68483.1		
Dermamoeba algensis		AY294148.1		
Dictyostelium discoideum			XP_637058.1	P54651
Eimeria acervulina			CAA61255.1	
Eimeria tenella			CAA61255.1	O44001
Entamoeba histolytica			P31017	XP_653132
Enteromonad	PSEUD	AY921408	EF551185	
Enteromonad	PYX	AY921407		
Enteromonadidae sp.	KR-PO3	AY701872.1		
Enteromonas hominis	ENTEROII	EF551180	EF551184	EF551168
Enteromonas sp.	GECA2	EF551178	EF551186	EF551169
Enteromonas sp.	CUORA1	EF551179		
Euglena gracilis			P33625	AAQ24862.1
Giardia ardeae		Z17210.1		
Giardia intestinalis		M54878.1	XP_001705720	BAD83616
Giardia intestinalis isolate BAC7		AF199444.1		
Giardia microti		AF006676.1		
Giardia muris		X65063.1		
Giardia sp.		U20351.1		
Guillardia theta		X57162.1		
Hexamita inflata		L07836.1	AAC47085.1	AAR26696
Hexamita nelsoni		EF050053.1		
Homo sapiens			NP_006073	NP031381
Ichthyobodo sp.		AY229972.1		
Jakoba libera		AF411288.1		
Macropharyngomonas halophila		AF011465.1		
Malawimonas jakobiformis		AY117420.1	AAK27846.1	ABC54645

Mesostigma viride		AJ250108.1		
Monocercomonas ATCC50210			AAD05022.1	
Monocercomonas ruminantium	KOJ14	AY319280.1		
Monocercomonoides sp.				AAW221773
Monosiga brevicollis		AF100940.1	AAK27410.1	AAP51213
Mus musculus			NP_035783.1	P11499
Naegleria gruberi		M18732.1	P11237	AAM937561
Neurospora crassa			XP_963223.1	XP323482
Noctiluca scintillans		AF022200.1		
Octomitus intestinalis		DQ366277.1		
Oryza sativa			NP_001051132.1	P33126
Paramecium tetraurelia			XP_001454509	AAG00568
Podocoryne carnea		AF358092.1		
Reclinomonas americana		AY117417.1		
Retortamonas sp.	KOZA1	AF439344.1		
Retortamonas sp.	LOS	AF439345.1		
Retortamonas sp.	OVCE	AF439346.1		
Retortamonas sp.	ATCC 50375	AF439347.1		
Retortamonas sp.	VALE	AF439348.1		EF551172
Saccharomyces cerevisiae			NP_013625.1	NP_015084
Schizosaccharomyces pombe		X58056.1		
Spironucleus salmonicida		DQ186595.1	AAC47212	ABC54647
Spironucleus meleagridis		EF050054.1		
Spironucleus muris		EU043230	AAC47088.1	
Spironucleus barkhanus		DQ186581.1	DQ181579	
Spironucleus salmonis		DQ394703.1		
Spironucleus sp.	GEPA2H	EF551181		
Spironucleus torosa		EF050055.1		
Spironucleus vortens		U93086.1	AAB81021.1	EF551170
Streblomastix strix			ABC97356.1	AAO46123
Tetrahymena thermophila			XP_001022424	AAD937561
Toxoplasma gondii			P10873.1	AAP44977
Trepomonas agilis		AF015455.1		
Trepomonas sp.	PPS6	EF551174		
Trepomonas steini	LUH3	EF551173		
Trichomonas vaginalis		U17510.1	AAK83156.1	XP_001317545
Trichonympha agilis		AB003920.1		
Trimastix marina		AF244905.1	ABC54661.1	ABC54648

Trimastix pyriformis		AF244903.1	PEP, Trimastix pyriformis Cluster TPL00000212	EU327684
Trimitus sp.	KOMPKOJ	EF551182		
Trimitus sp.	IT1	EF551176		
Trimitus sp.	DOGA1	EF551177		
Trimitus sp.	RAPI1	AY701873.1		
Trimitus sp.	TRION	AY701874.1	EF551183	EF551171
Trypanosoma cruzi			XP_802499.1	A26125
Uncultured eukaryote	CHESI2	EF551175		
Uncultured *Oxymonas* sp.		AB092931.1		
Volvox carteri		X53904.1		
